# Editorial: Exploring the interplay between stress, neuropeptides, and alcohol use disorder

**DOI:** 10.3389/fphar.2026.1930157

**Published:** 2026-07-24

**Authors:** Kabirullah Lutfy, Marcelo López

**Affiliations:** 1 Department of Biotechnology and Pharmaceutical Sciences, College of Pharmacy, Western University of Health Sciences, Pomona, CA, United States; 2 Departments of Neuroscience and Psychiatry, Charleston Alcohol Research Center, Medical University of South Carolina, Charleston, SC, United States

**Keywords:** alcohol use disorders, neuropeptides, calcitonin gene-related peptide, dynorphin, neuropeptide Y, orexin, receptors, stress

Alcohol use disorders (AUD) are major public health issues, with a significant negative impact on the health system and the economy. Unfortunately, only a handful of pharmacotherapeutic agents are available to treat patients with AUD. Notably, these medications have limited efficacy in reducing alcohol consumption, craving, and relapse. Therefore, there is a need for development of more effective medications to treat AUD. Preclinical studies as well as some clinical trials have shown that a series of neuropeptides, such as dynorphin, orexin, etc., as well as peptides released from the periphery and acting centrally, such as glucagon-like peptide, amylin, and ghrelin, have shown promise to alter alcohol self-administration, signs of withdrawal, and relapse. These peptides also show promise in reducing stress-mediated craving and relapse, which is a central issue in treating alcohol use disorders. In this Research Topic series, Holguin and Martin-Fardon provide a review of the literature on the interaction between orexin (OX) and dynorphin (DYN) in the posterior paraventricular nucleus of thalamus (pPVT) and postulate that this interaction may underly the stress-mediated craving and relapse after chronic alcohol use. These authors also discuss the potential of targeting the receptors for each peptide to reduce stress-induced alcohol craving and relapse. They also propose that a deeper understanding of the balance between the two neuropeptide systems within the reward and stress circuits may unveil insights for relapse prevention in individuals with AUD. In an elegant study, Van Doorn et al. determined the role of calcitonin gene-related peptide in the parabrachial nucleus (PBN) in anxiety-like behaviors in mice undergoing abstinence, as the level of CGRP is elevated in alcohol-drinking and alcohol-preferring subjects. The authors focused on PBN as it contains high levels of CGRP. Calca-cre mice and Designer Receptor Exclusively Activated by Designer Drugs (DREADDs) were used to assess the role of PBN CGRP in anxiety-like behaviors following alcohol withdrawal compared to naïve controls. The authors proposed that PBN CGRP may be a potential target to treat anxiety-like behaviors that develop following acute withdrawal. In another study, Flores-Ramirez et al. determined the role of DYN and OX in the pPVT in alcohol reinstatement using the operant conditioning paradigm. Rats were trained to self-administer a 10% ethanol solution for 3 weeks followed by a 6-week chronic intermittent alcohol vapor exposure. Rats underwent extinction training and then tested for stress-induced reinstatement of alcohol self-administration following local intra-pPVT OX or kappa opioid receptor (KOR) antagonist or their combination. While either antagonist reduced reinstatement of ethanol self-administration, their combination was not as effective. An increase in the level of both peptides was observed in the hypothalamus along with an increase in KOR gene (Oprk1) and decrease in OX receptor 1 gene (Hcrt1) were also observed in the pPVT, showing an interaction between the two peptides/receptor systems in the pPVT. The study by Zhao et al. adds relevant evidence on the effect of a history of alcohol consumption on stress reactivity. Using a model of voluntary alcohol intake in mice combined with a single prolonged stress procedure that results in PTSD-like symptoms, the study evaluates whether activation of cyclic adenosine monophosphate/protein kinase A (cAMP-PKA) pathway can be manipulated to attenuate the effects of alcohol on stress responsiveness. Their data show that activation of this pathway, which results in modulation of growth factors, represents a promising alternative to attenuating PTSD-like symptoms, particularly in subjects with a history of AUD.

Overall, the articles included in this Research Topic offer new relevant evidence of the role of neuropeptides modulating circuitry associated with stress and negative affect that can help perpetuate problem drinking and/or relapse. Hopefully, this will foster further development in this area to evaluate promising new medications to treat AUD.

